# Supplementary material for: Modeling glioblastoma heterogeneity as a dynamic network of cell states
Source: Mol Syst Biol. 2021 Sep 16;17(9):e10105. doi: 10.15252/msb.202010105 (PMC8444284; doi:10.15252/msb.202010105)
Supplement: Supplementary file 6 — Source Data for Figure 5 [file MSB-17-e10105-s004.zip › Figure5A_sourcedata/GSEA_3071/hallmarks_stateIII.GseaPreranked.1623742540963/HALLMARK_COMPLEMENT.html]

Details for gene set HALLMARK\_COMPLEMENT[GSEA]

|  || Dataset | state33071 |
| Phenotype | NoPhenotypeAvailable |
| Upregulated in class | na\_neg |
| GeneSet | HALLMARK\_COMPLEMENT |
| Enrichment Score (ES) | -0.26073116 |
| Normalized Enrichment Score (NES) | -1.4112008 |
| Nominal p-value | 0.101528384 |
| FDR q-value | 0.21877107 |
| FWER p-Value | 0.988 |
Table: GSEA Results Summary

  

Fig 1: Enrichment plot: HALLMARK\_COMPLEMENT      
 Profile of the Running ES Score & Positions of GeneSet Members on the Rank Ordered List

  

| PROBE | GENE SYMBOL | GENE\_TITLE | RANK IN GENE LIST | RANK METRIC SCORE | RUNNING ES | CORE ENRICHMENT || 1 | PFN1 |  |  | 40 | 0.481 | 0.0124 | No |
| 2 | CALM1 |  |  | 209 | 0.334 | -0.0151 | No |
| 3 | ATOX1 |  |  | 233 | 0.321 | -0.0059 | No |
| 4 | CSRP1 |  |  | 345 | 0.279 | -0.0214 | No |
| 5 | CTSL |  |  | 492 | -0.254 | -0.0471 | No |
| 6 | CTSO |  |  | 615 | -0.260 | -0.0662 | No |
| 7 | CPM |  |  | 637 | -0.261 | -0.0593 | No |
| 8 | MAFF |  |  | 735 | -0.266 | -0.0717 | No |
| 9 | GNB4 |  |  | 885 | -0.274 | -0.0972 | No |
| 10 | CD46 |  |  | 1039 | -0.281 | -0.1234 | No |
| 11 | TFPI2 |  |  | 1128 | -0.286 | -0.1326 | No |
| 12 | SIRT6 |  |  | 1138 | -0.287 | -0.1214 | No |
| 13 | JAK2 |  |  | 1401 | -0.300 | -0.1748 | No |
| 14 | CTSC |  |  | 1511 | -0.306 | -0.1884 | No |
| 15 | ZEB1 |  |  | 1569 | -0.310 | -0.1885 | No |
| 16 | CDK5R1 |  |  | 1602 | -0.312 | -0.1820 | No |
| 17 | ZFPM2 |  |  | 1615 | -0.313 | -0.1703 | No |
| 18 | ERAP2 |  |  | 1836 | -0.326 | -0.2117 | No |
| 19 | ADAM9 |  |  | 1870 | -0.328 | -0.2047 | No |
| 20 | AKAP10 |  |  | 1935 | -0.331 | -0.2055 | No |
| 21 | LAMP2 |  |  | 1956 | -0.333 | -0.1950 | No |
| 22 | TIMP1 |  |  | 2038 | -0.337 | -0.2000 | No |
| 23 | CTSD |  |  | 2110 | -0.342 | -0.2021 | No |
| 24 | PLAUR |  |  | 2181 | -0.347 | -0.2038 | No |
| 25 | MSRB1 |  |  | 2403 | -0.365 | -0.2435 | Yes |
| 26 | CTSS |  |  | 2434 | -0.368 | -0.2338 | Yes |
| 27 | SH2B3 |  |  | 2459 | -0.370 | -0.2226 | Yes |
| 28 | PRCP |  |  | 2489 | -0.372 | -0.2125 | Yes |
| 29 | BRPF3 |  |  | 2672 | -0.388 | -0.2411 | Yes |
| 30 | CBLB |  |  | 2745 | -0.394 | -0.2410 | Yes |
| 31 | HSPA1A |  |  | 2774 | -0.396 | -0.2295 | Yes |
| 32 | MMP14 |  |  | 2811 | -0.400 | -0.2199 | Yes |
| 33 | CTSB |  |  | 2916 | -0.410 | -0.2274 | Yes |
| 34 | RCE1 |  |  | 2973 | -0.417 | -0.2222 | Yes |
| 35 | TNFAIP3 |  |  | 3088 | -0.430 | -0.2313 | Yes |
| 36 | HSPA5 |  |  | 3193 | -0.442 | -0.2372 | Yes |
| 37 | GATA3 |  |  | 3260 | -0.453 | -0.2329 | Yes |
| 38 | C1R |  |  | 3364 | -0.473 | -0.2371 | Yes |
| 39 | PCLO |  |  | 3417 | -0.483 | -0.2277 | Yes |
| 40 | SERPINE1 |  |  | 3474 | -0.499 | -0.2186 | Yes |
| 41 | PDP1 |  |  | 3555 | -0.528 | -0.2143 | Yes |
| 42 | LRP1 |  |  | 3584 | -0.540 | -0.1960 | Yes |
| 43 | PLAT |  |  | 3600 | -0.547 | -0.1740 | Yes |
| 44 | DUSP6 |  |  | 3648 | -0.577 | -0.1589 | Yes |
| 45 | CFH |  |  | 3653 | -0.582 | -0.1325 | Yes |
| 46 | CDH13 |  |  | 3656 | -0.583 | -0.1055 | Yes |
| 47 | COL4A2 |  |  | 3664 | -0.586 | -0.0796 | Yes |
| 48 | F3 |  |  | 3764 | -0.693 | -0.0724 | Yes |
| 49 | PDGFB |  |  | 3803 | -0.751 | -0.0468 | Yes |
| 50 | CLU |  |  | 3846 | -0.827 | -0.0186 | Yes |
| 51 | FN1 |  |  | 3850 | -0.842 | 0.0204 | Yes |
Table: GSEA details [plain text format]

  

Fig 2: HALLMARK\_COMPLEMENT: Random ES distribution      
 Gene set null distribution of ES for **HALLMARK\_COMPLEMENT**
